# Clinical Microbiology: where do we stand?

**DOI:** 10.3389/frabi.2024.1250632

**Published:** 2024-06-17

**Authors:** Alkiviadis Vatopoulos

**Affiliations:** Department of Public Health Policy, School of Public Health, University of West Attica, Athens, Greece

**Keywords:** Clinical Microbiology, health technology assessment (HTA), next-generation sequencing (NGS), polymerase chain reaction (PCR), future developments in microbiology

## Abstract

Clinical Microbiology has developed during the last 100 years, simultaneous with the discovery of microorganisms as causes of infections. Globalization and One Health determine present needs whereas molecular biology, automation, artificial intelligence, and bioinformatics are new tools that characterize the new developments in the field.

## Introduction

Clinical Microbiology deals with the laboratory diagnosis of infections in humans and animals. This is done through the isolation and identification of the causative organisms, including viruses, bacteria, fungi, and parasites, by culturing or by isolating the genetic material in the human host, or through the identification of the specific immune reaction the causative organism produces. Microbiology also contributes to the treatment of infections through the determination of sensitivity of the causative organisms to antibiotics, the understanding of the spread of microorganisms in human and animal populations, the emergence and reemergence of new pathogens, and the identification and understanding of possible new infectious diseases.

Clinical Microbiology, as a discipline, emerged simultaneously with the discovery of microorganisms as causative agents of infectious diseases at the end of the 19th century (de Kruif, 1927).

Methods of culturing infectious microorganisms developed through the understanding of their biology and the development of various specific culture media. Methods of identification developed exploiting their physical and biochemical characteristics. Flow charts aid the work of the clinical microbiologist in the lab, whereas numerical taxonomy is developed and facilitated in everyday practice (Carroll and Pfaller, 2023).

The study and understanding of the phenotypic, and later, the genotypic characteristics of organisms, to comprehend pathogenesis and to develop the necessary diagnostic techniques, are also areas of Clinical Microbiology.

Microorganisms are classified into taxa, genera, and species, based mainly on the similarity of their physical and biochemical characteristics because taxonomy based on the phylogeny of the microorganisms was not possible. However, the development of molecular biology and the understanding of the role of the ribosomal DNA as the genetic clock are now used to further classify microorganisms genetically (Woese, 1987).

Gram stain and other bacteriological stains on the clinical sample, as well as the use of the principle of the antigen antibody reaction (ELISA, RIA, and IF) are the main techniques used in the clinical laboratory for the direct identification of pathogens (Carroll and Pfaller, 2023).

Culturing the clinical sample on selective media and studying the physical and biochemical reactions of the isolated organisms are the main techniques for the isolation and study of pathogens. The use of chromogenic agars is a recent development (Perry, 2017). They contain a chromogenic substrate that is split by an enzyme characteristic of the target organism into a sugar component and a chromogen. In the presence of oxygen, the chromogen forms a dimer that colors the broth or the typical colony.

The antibiogram based on the disk diffusion method or the determination of the minimum inhibitory concentration (MIC) of the organisms is a widely utilized methodology for studying the sensitivity and resistance of the pathogen to antibiotics. Although broth macrodilution is the reference method for MIC determinations, broth microdilution with the use of commercially available systems is established in most clinical laboratories. Disk diffusion is cheap and flexible but demanding in workload and expertise, whereas MIC microdilution is more expensive and less flexible (a standard combination of antibiotics is used) but can be automated and demands less expertise. ETest is a recent development in technology for MIC determinations in the clinical laboratory, making the technique more flexible in the selection of antibiotics to be used. ETest consists of a predefined gradient of antibiotic concentrations immobilized on a plastic strip and is used to determine the MIC of antibiotics and antifungal agents (Kahlmeter and Brown, 2010). Detecting resistance mechanisms by observing synergy or antagonism among antibiotics in the antibiogram based mainly on the disk diffusion method, a technique known as the interpretive reading of the antibiogram has resulted in the development of expert rules that are increasingly used in the lab (https://www.eucast.org/expert_rules_and_expected_phenotypes). Lateral flow tests (monotests) have also been developed for detecting resistance mechanisms, extremely useful in screening and epidemiology (Boutal et al., 2022). Concerning antiviral testing, the technical challenges of NGS, such as polymerase chain reaction (PCR) and sequence-related errors, are being addressed and various assays are currently undergoing technical validation for clinical use (Smit, 2014).

Moreover, the study of the immunology of infection is an important area of Clinical Microbiology, and various assays based on the specific antibody to antigen reaction are used to identify pathogens in the clinical sample. Lateral flow tests (monotests) such as *Strept test* for the diagnosis of Streptoccocal infections and the detection of HBsAg are common examples of this approach. Moreover, the immunological reaction of humans to microorganisms is being exploited in the lab to identify the presence of specific antibodies and thus, the presence of infection, and the respective techniques are continuously under development.

Typing of organisms is also a crucial area in Clinical Microbiology (Sabat et al., 2013), for understanding the epidemiology of infections and thus, for elucidating and combating epidemics. Serotyping is a classic phenotypic technique, whereas molecular techniques are now the most in-use methods. Pulse field gel electrophoresis is a standard technique used especially in hospital epidemiology (Neoh et al., 2019), whereas sequencing techniques such as multilocus sequence typing are the molecular approach for understanding the global epidemiology of infections such as Pneumococcal infections or gonorrhea (Maiden et al., 1998; de Sales et al., 2020). User-friendly bioinformatics software solutions for analyzing the increasing volume of data are also increasingly used (as an example, https://www.bionumerics.com/applications). This is currently done mainly in reference centers, however, clinical laboratories must have access to their results, and more importantly, must have the knowledge to interpret them.

Increasing automation is also characteristic of the modern clinical laboratory. This automation includes the pre-analytic phases, with barcoding of the samples and distributing them to analyzers up to the automatic download of the results in the information system of the lab (LIS) or the hospital.

Although culturing of clinical samples continues to be done manually, automatic analyzers have been developed for processing clinical specimens and performing traditional microbiology techniques including blood culturing, bacterial identification, and sensitivity testing. These analyzers include large databanks of antibiograms. They perform routine and more sophisticated hospital epidemiological studies and can download data to national and international epidemiological systems including systems for antibiotic resistance surveillance.

Most immunology tests are also performed on automatic analyzers using traditional ELISA assays as well as more sophisticated assays such as enzyme-linked fluorescent assay. Immunofluorescence assays are also becoming increasingly automated.

All these analyzers have as common characteristics are user friendliness and embedded quality control systems to maximize efficiency and to minimize the possibility of error.

However, an important side effect of automation is the “black box effect” in the lab: The result of the test is the result of the automatic analyzer, without any real knowledge of the respective biology. An isolate is *Klebsiella pneumoniae* because the “machine says so”, and not as a result of the work out of the organisms. There is a gap between the procedures in the lab and the scientists working there. In that respect, continuous training of the scientific and technical staff in the lab is very important for the understanding of the logic of automation and the biological basis of the diagnostic procedures.

Innovative techniques, including mass spectrometry are increasingly in use in the modern lab. Matrix-assisted laser desorption/ionization time of fight (MALDI-TOF) is the main example in the use of this technique for bacterial identification and typing (Singhal et al., 2015).

Another important development within the laboratory is the increasing use of molecular techniques for the isolation and identification of infectious agents, as well as in the identification of antibiotic resistance genes. Although culture and antibiotic sensitivity testing continue to be the gold standards in Clinical Microbiology, PCR with the use of universal primers and sequencing of the product is increasingly used for isolation and identification in the everyday laboratory practice. Quantitate PCR (rtPCR) is an important tool for quantification of nucleic acids, and thus, of the infectious agent in clinical specimens.

Molecular techniques are crucial for the diagnosis of infections where classic techniques are time-consuming (for example, tuberculosis), or in cases where there is no alternative (COVID-19 or hepatitis C virus). Microarray analysis, which can offer robust multiplex detection, has also entered the diagnostic microbiology laboratory and offers multiplex detection and characterization for a variety of infectious disease pathogens. However, it must be understood that PCR detects genetic material and not necessarily a viable infectious organism. In that respect, techniques to detect messenger RNA, which can suggest viability, might be a development in this area.

The increasing development and use of molecular biology techniques combined with the simultaneous development of specific analyzers have made virology, an area limited mainly to research or reference labs, incorporated into most standard clinical laboratories. Virology is an area of intensive research for the development of new diagnostic techniques.

Recent experience has shown the necessity for laboratories to be able to rapidly adjust in the use of diagnostic tests for new diseases that have a high social impact, as was the case with the outbreak of severe acute respiratory syndrome (SARS) and, more recently, COVID-19.

Next step in the use of molecular biology in Clinical Microbiology will be the adaptation of whole genome sequencing techniques such as next-generation sequencing (NGS), which is a massively parallel sequencing technology that offers ultra-high throughput, scalability, and speed (Behjati and Tarpey, 2013), or deep sequencing (Goldman and Domschke, 2014), which refers to sequencing a genomic region multiple times, sometimes hundreds or even thousands of times. This NGS approach allows researchers to detect rare clonal types, cells, or microbes comprising as little as 1% of the original sample for detecting and identifying pathogens in everyday laboratory practice.

Analyzers based on molecular biology techniques are also developed. These analyses include sufficient quality control procedures, and their operations are simple enough to be used by non-specialized lab technicians.

Accreditation of the lab through the main ISO protocols to safeguard quality, which includes internal and external quality control schemes, is now necessary in most health systems throughout the world.

The increasing cost of new tests has also resulted in the centralization and merging of clinical laboratories: Large central labs have been developed for the service of many hospitals and outpatient clinics, and procedures for the safe transfer of samples have also been developed. A side effect of this trend is the increasing lack of communication between the lab and clinicians, the lab being more technical and less clinical. Video conferencing, increasingly in use since the COVID-19 pandemic might be a way to deal with this.

Hospital-acquired infections and bacterial antibiotic resistance are important areas of Clinical Microbiology. The increasing prevalence of multidrug resistant pathogens in hospitals and the difficulties in their prevention and treatment have increased the need for meticulous study in areas such as pathogenesis and bacterial pathogenetic mechanisms, diagnosis and treatment including sensitivity testing, and understanding of the development and spread of Multi Drug Resistant (MDR) bacteria in the hospital.

The impressive development in medicine during the last decades has resulted in the extension of expected lifespan of patients with serious conditions including chronic disease with immunosuppression and consequently increased liability to infections. This has also increased the need for research in this area, including newer pathogens in immunosuppressed patients, diagnostics and therapy. It is well understood that microorganisms regarded as nonpathogenic decades ago, including *Acinetobacter* spp. and coagulase-negative Staphylococci, are now major pathogens in Hospitals. In that respect, the lab must constantly adjust to be able to identify these organisms and to contribute to the understanding of their role. A quite common and crucial clinical question that must be answered in the everyday clinical practice is whether “the patient died *due* to infection, for example by *Acinetobacter baumannii* or being colonized by *Acinetobacter baumannii*.”

Developing screening strategies for patients at entry for carriage of multiresistant organisms is another important task of the clinical laboratory in the hospital, as well as the routine screening of immunocompromised patients and for patients in the ICU.

Hospital epidemiology is also based on the data of the clinical laboratory, and the quality of these data as well as the quality of the collaboration among the lab and the epidemiologists are crucial for early diagnosis of possible hospital epidemics.

Globalization and the extensive international movements of humans, animal, and goods have resulted in the international spread of infectious agents. Emerging and reemerging diseases (EID) are an important area of Clinical Microbiology. Interestingly, events due to emerging and reemerging diseases are dominated by zoonoses (60.3% of EIDs): The majority of these (71.8%) originate in wildlife, for example, severe acute respiratory virus, Ebola virus), and are increasing significantly over time (Jones et al., 2008). In that respect, Clinical Microbiology is affected by the One Health approach to health. Close collaboration with veterinary medicine is important for the diagnosis and understanding of zooanthroponosis, infections due to pathogens reservoir in humans that are capable of being transmitted to other non-human animals and vice versa. The development of a common language between the two disciplines is crucial.

Clinical medicine all over the world must have the readiness to include in the differential diagnosis infections that are not routinely found in its geographic area. West Nile virus is an example of a disease that due to the recent mobility of its vector (mosquito Culex), resulted in epidemics in Europe and the USA, and the urgent need for the respective development of diagnostic capacity. Similarly, HIV, SARS, MERS, bird flu, monkeypox, and SARSCOV-2, are examples of emerging pathogens that potentially resulted in the development of epidemics or pandemics.

In particular, HIV, with an array of opportunistic infections HIV patients developed, resulted in an increase in the diagnostic capacity of the clinical laboratory to include an array of, up to that point, rare pathogens such as *Coccidioidesimmitis*, *Coccidioides posadasii, Pneumocystis jirovecii, Histoplasma capsulatum, and Encephalitozoon bieneusi*.

The last few decades have been characterized by an increase of migration including refugees and financial migrants, especially in Europe and North America. The estimated number of international migrants has increased over the past five decades. The total estimated 281 million people living in a country other than their countries of birth in 2020 was 128 million more than in 1990, and over three times the estimated number in 1970. Infectious diseases among migrants are known to have a negligible impact on European or North American epidemiology. However, screening programs need to be implemented and adapted to the different stages of the migratory process to better understand the trends and set priorities for action (Castelli and Sulis, 2017; Vignier and Bouchaud, 2018). Moreover, infections that are considered tropical and rare in the West must increasingly be included in differential diagnosis. Respectively, the clinical laboratory must have the expertise and resources to diagnose these infections or have in place, a strategy through the development of networks with reference labs to fulfill the need. Their conformation was made possible through the early development of diagnostic techniques and infrastructure as well as the establishment of networks through national or international cooperation.

Intensive farming and the urbanization of the forests have increased the “spillover” of zoonotic agents to humans that must be confronted by the scientific communication and combined research between human and veterinary Clinical Microbiology as well as between Clinical Microbiology and environmental microbiology (Carlson et al., 2022; Li et al., 2023).

Lastly, the aging of the population and the increasing number of immunocompromised patients that survive due to the recent developments in medicine, are also causes for the increased incidence of infections, especially of emerging and reemerging organisms (Gavazzi and Krause, 2002; El Chakhtoura et al., 2017).

In these situations, emerging and reemerging diseases must be recognized and studied, and new laboratory techniques must be developed and evaluated for their diagnosis and clinical evaluation.

The recent developments in areas such as computer sciences and molecular biology have increased research on their possible use in Clinical Microbiology. This includes methods of analyzing Big Data, now accumulated in the clinical laboratory through machine learning that they have focused on classification problems and analysis of interaction problems (Qu et al., 2019). The use of artificial intelligence in the development and validation of molecular diagnostics, in particular, the use of NGS in clinical practice (Gargis et al., 2016), the development and validation of point-of-care testing (POCT), and the development and validation of new molecular diagnostic assays needs to be studied. The use of automation in the Clinical Microbiology laboratory is also an area of active research as well as financial issues including the cost–benefit analyses of the use of the various laboratory techniques, and financial aspects in laboratory practice, including the health technology assessment (HTA) approach. In particular, HTA in Clinical Microbiology involves the evaluation of the value, safety, and efficiency of various technologies [as an example, see (Stevenson et al., 2016)]. In that respect, HTA plays a crucial role in the decision-making process of the adoption and utilization of microbiological tests and procedures, use of antimicrobial agents, and epidemiological and infection control measures for the more efficient and effective use of the laboratory data for better treatment of patients, and ultimately, decrease of the length of stay in the hospital and increase turnaround time.

In summary, the increase in the prevalence, importance, and variety of infections has amplified the significance of Clinical Microbiology as a medical specialty, to include molecular biology, informatics, and artificial intelligence as important tools in research, also for improving everyday function in the clinical laboratory. Accreditation has increased efficiency, quality, and credibility; however, centralization and automation could act against scientific collaboration in the hospital. Concerning management issues, the HTA approach is increasingly used in the laboratory, and continuous training in the clinical laboratory must be an integral part of everyday routine.

## Author contributions

AV: Writing – original draft, Writing – review & editing.
